# Surgical navigation improves reductions accuracy of unilateral complicated zygomaticomaxillary complex fractures: a randomized controlled trial

**DOI:** 10.1038/s41598-018-25053-z

**Published:** 2018-05-02

**Authors:** Xiao Zhang, Lanfeng Ye, Hui Li, Yi Wang, Dilnur Dilxat, Weilong Liu, Yuanwei Chen, Lei Liu

**Affiliations:** 1 Department of Oral & Maxillofacial Surgery, West China Hospital of Stomatology, Sichuan University, Chengdu, 610041 P.R. China; 2 Key Laboratory of Oral Medicine, Guangzhou Institute of Oral Disease, Stomatology Hospital of Guangzhou Medical University, Guangzhou, 510140 China

## Abstract

Accurate reduction is the key to successful treatment of bone fractures. Complicated zygomaticomaxillary complex fracture, known as one of the most challenging facial bone fractures, is often hard to achieve an accurate reduction, thus leading to facial deformity. In this study, twenty patients with unilateral complicated zygomaticomaxillary complex fractures were included and randomly divided into experimental and control groups, which is with and without the aid of surgical navigation, respectively. The pre- and postoperative imaging data were collected and then analysed using Geomagic Studio 11 software and Brainlab iPlan CMF 3.0. A more precise reduction was showed in the experimental group according to the measurement results of both software programmes than in the control group. In conclusion, surgical navigation showed great value in performing accurate reductions of complicated zygomaticomaxillary complex fractures and restoring facial contour.

## Introduction

The face occupies the most prominent position in the human body, rendering it vulnerable to injuries quite commonly^1,2^. Among facial fractures, the zygomaticomaxillary complex fracture is one of the most frequently occurring^3^. Patients suffering from zygomaticomaxillary complex fractures always present deformity because the zygomaticomaxillary complex strongly contributes to midfacial width and protrusion^3,4^. Hence, accurate reductions of zygomaticomaxillary complex fractures play a vital role in re-gaining satisfactory facial contour^5^. For invert fractures, open reduction with internal fixation (ORIF) should be considered as the main and reliable option for restoring appearance^6,7^. However, fractures are quite difficult to handle, especially in cases of complicated zygomaticomaxillary complex fractures. Complicated zygomaticomaxillary complex fractures refer to comminuted fractures, fractures with delayed surgery and/or bone defects of the zygomaticomaxillary complex. Lacking available landmarks for anatomical reduction, surgeons handle the fractured fragments mainly according to their own clinical experience, thus frequently resulting in unsatisfactory outcomes of over- or under-reduction^7,8^. Failure of anatomical reduction will cause the incorrect width and protrusion of zygoma and asymmetry of midface, and may lead to poor self-esteem and health problems related to poor quality-of-life. Therefore, an accurate reduction is the key to the treatment of complicated zygomaticomaxillary complex fractures^9–11^.

With the rapid development of computer technology, surgical navigation has gradually become a new supportive tool for diagnosis, operation planning and treatment in medicine^9,12^. For synchronization of real-time actual surgical anatomy with the imaging of the patient’s anatomy previously obtained by CT imaging, surgical navigation was first used in neurosurgery and has gained rapid progress^12,13^. In recent years, surgical navigation has been applied in many surgical procedures, such as foreign body removal, tumour resection, deformity correction, and implantation^14–17^. These studies indicated that with the assistance of surgical navigation, precise and predictable results were achieved. Whether surgical navigation really improves the accuracy of reductions of complicated zygomaticomaxillary complex fractures is still controversial^18^. In this study, a randomized controlled clinical trial was conducted to compare the treatment effects of complicated zygomaticomaxillary complex fractures with and without the surgical navigation. Planning software was used to simulate surgical movements of fractured bones based on preoperative images, and intraoperative surgical navigation was used to assist the accurate reduction. In conclusion, we found surgical navigation for the treatment of zygomaticomaxillary complex fractures can lead to significant improvements in the accuracy of reductions, which should become an indispensable part of surgical therapy.

## Results

Twenty patients with unilateral complicated zygomaticomaxillary complex fractures were included and divided into experimental and control groups in the study, including 13 men and 7 women, with a mean age of 34.55 years old (range, 18 to 58). The causes of the injury were followed by: traffic accidents, 10 (50%); fall damage, 5 (25%); violent injury, 3 (15%); explosive injury, 1 (5%); bear scratch, 1 (5%). All the patients involved in were treated by ORIF and completed follow-up procedures. Clinical characteristics of patients were showed as follows (Table 1).

**Table 1:** Comparison of clinical characteristics of patients between the two groups.

Characteristics		Group		P-value
		Experimental	Control	
Age(in years)	N	10	10	NA
	Mean ± SD	33.60 ± 11.36	35.50 ± 11.39	0.70
Gender	N (Women)	3	4	0.50
	N (Men)	7	6	
Maximum mouthopening (mm)	Mean ± SD	33.20 ± 2.23	32.50 ± 2.06	0.50
Diplopia	N (completely resolved)	2	2	0.60
	N (partially resolved)	0	1	
Fracture reduction	N (Anatomic and approximately anatomic)	9	8	0.39
	N (Nonanatomic)	1	2	
Wound Infection	N	0	0	NA
Surgical approaches	N (hemicoronary and intraoral vestibule incisions)	8	7	0.35
	N (hemicoronary and intraoral vestibule incisions combined subconjunctival incisions and/or facial scar)	2	3	
Time(minutes)	Mean ± SD (preoperative plan)	48.00 ± 8.31	46.80 ± 9.69	0.78
	Mean ± SD (surgery)	183.70 ± 25.33	179.00 ± 21.15	0.30

Using the independent-sample t-test for comparison of the postoperative maximum mouth opening and time. Using a chi-squared test (Fisher exact probability method) for comparison of the other variables; P value of <0.05 was considered to indicate a statistically significant difference.

### Outcomes of primary variable

The measurement results of Geomagic Studio 11 software (Geomagic, NC State, USA)were shown as follows. The average distance (AD) of landmarks was 0.593 (0.427 to 0.834) mm in the experimental group and 1.233 (0.735 to 1.583) mm in the control group (P < 0.01). The results indicated that in the experimental group, the postoperative position of the fractured bone was more consistent with the ideal position reconstructed preoperatively than the control group (Table 2).

**Table 2:** Comparison of AD of landmarks(mm).

Group	Numberof patients	Mean	SD	Minimum	P25	Median	P75	Maximum	t	P-value
Experimental	10	0.59	0.14	0.37	0.49	0.62	0.66	0.83	−6.61	1.65*10^−6^
Control	10	1.23	0.27	0.74	1.16	1.24	1.43	1.58		

Using the independent-sample t-test for comparisons; the statistical magnitude was t.

The measurement results of the Brainlab iPlan CMF 3.0 software (Brainlab, Heimstetten, Germany) were shown as follows. Surgical deviation in the intersection point of the zygomaticomaxillary suture and the infraorbital margin (oz) was 0.69 (0.40 to 1.40) mm in the experimental group and 1.12 (0.60 to 1.50) mm in the control group (P < 0.01). Surgical deviation in the most inferior point of the temporozygomatic suture (ztl) was 0.87 (0.40 to 1.50) mm in the experimental group and 1.57 (0.50 to 3.50) mm in the control group (P < 0.05). The surgical deviation in the most anterior point of the zygoma (mp) was 0.98 (0.20 to 2.00) mm in the experimental group and 1.22 (0.50 to 2.00) mm in the control group (P > 0.05). Surgical deviation in the most lateral point of the front zygomatic suture (fmt) was 0.68 (1.20 to 2.60) mm in the experimental group and 0.81 (0.40 to 1.00) mm in the control group (P > 0.05). The results indicated that for the landmark oz and ztl, the reduction accuracy in the experimental group was better than the control group (Table 3).

**Table 3:** Comparison of surgical deviation of landmarks (mm).

Landmark	Group	Numberof patients	Mean	SD	Minimum	P25	Median	P75	Maximum	t	P-value
mp	Experimental	10	0.98	0.50	0.20	0.65	1.00	1.20	2.00	−1.78	0.127
	Control	10	1.22	0.41	0.50	1.00	1.25	1.45	2.00		
fmt	Experimental	10	0.68	0.51	0.20	0.23	0.60	0.88	1.60	−0.76	0.227
	Control	10	0.81	0.17	0.40	0.80	0.85	0.90	1.00		
oz	Experimental	10	0.69	0.37	0.40	0.43	0.65	0.90	1.40	−2.91	0.005
	Control	10	1.12	0.28	0.60	1.00	1.15	1.35	1.50		
ztl	Experimental	10	0.87	0.33	0.40	0.65	0.85	1.00	1.50	−2.54	0.010
	Control	10	1.57	0.81	0.50	1.20	1.40	1.35	3.50		

Using the independent-sample t-test for comparisons; the statistical magnitude was t.

Combining the results of data analysed by the Geomagic Studio 11 software and Brainlab iPlan CMF 3.0 software, we draw the conclusion that the reduction accuracy in the experimental group was preferable than that in the control group.

### Outcomes of other variables

All patients achieved satisfactory results in two weeks after operation. No wound infection or other severe complications occurred in any patient. There was no looseness and migration of titanium plates and titanium screws, verified by imaging tests. In addition, there was no infection or intracranial injury resulting from the installation of the navigation reference frame in the experiment group. All the scars within the hairline were invisible and acceptable.

## Discussion

The zygomaticomaxillary complex, a major component of the midface, determines the midfacial width and protrusion^16,19^. Once zygomaticomaxillary complex fractures occur, midfacial deformities are mostly involved. Therefore, the primary treatment objective of zygomaticomaxillary complex fractures is restoration of the facial contour, and the key to treatment is the reduction accuracy. However, reduction is still greatly dependent at present on the preference and experience of surgeons, make it hard for beginning and inexperienced surgeons to achieve anatomic reductions. Once complicated zygomaticomaxillary complex fractures occur, accurate reduction will be a daunting challenge even for experienced surgeons. Thus, complicated zygomaticomaxillary complex fractures are always regarded as one of the most challenging facial bone fractures to treat^2,8,20^.

Surgical navigation provides new ideas and means to help solve this problem. Previous studies reported that surgical navigation was used to assist in treating zygomaticomaxillary complex fractures^13,14,21–24^. Carsten Westendorff *et al*.^21^ conducted a non-comparative pilot study of five patients with severely displaced orbitozygomatic fractures. All patients in the study were treated with surgical navigation and got satisfactory results. Akihiro Ogino *et al*.^5^ showed that surgical navigation assisted in improving accuracy of the reduction and achieving recovery of the profile after treating six patients with zygomatic fractures. But to date, most of the studies were case reports or clinical experience lacking multiple variables and randomized controlled design, which makes the effects of treatment less persuasive^13,19,25,26^. Whether surgical navigation really improves the accuracy of reductions in the treatment of complicated zygomaticomaxillary complex fractures remains controversial. Some scholars think surgical navigation is an effective aided tool, while others think it is just an expensive toy^14,19^. Therefore, we conducted this randomized controlled clinical trial and twenty patients were randomly assigned to the experimental group and the control group to make an objective assessment of the effects of the surgical navigation technique.

To make the results more objective and valid, two software programmes were applied to evaluate the value of the surgical navigation technique in the reduction and fixation of unilateral complicated zygomaticomaxillary complex fractures. The results of the mean values of AD measured by the Geomagic Studio 11 software in two groups were, respectively, 0.59 (0.74–1.58) mm and 1.23 (1.94–2.56) mm. The AD in the experimental group was significantly lower than in the control group. And the mean values of surgical deviations measured by the Brainlab iPlan CMF 3.0 software in the two groups were, respectively, landmark oz (0.69 mm vs 1.22 mm) and landmark ztl (0.87 mm vs 1.57 mm). The mean value of surgical deviations of the landmark oz and ztl was lower in the experimental group. The above results indicated that surgical navigation could assist with achieving better accuracy. Surgical navigation can assist the accurate locations of the surgical instruments and the surrounding anatomic structure in real-time, can guide to complete reductions and can check visually whether the position of the moved segments is desired, which makes the surgery precise, minimal and less invasive.

For the evaluation of treatment outcomes of the complicated zygomaticomaxillary complex fractures, there have been no standardized methods. At present, the following methods are commonly used: (1) The patients’ feedback. In general, if the clinical symptoms caused by the fractures completely resolve or partially resolve after surgery, patients will think that they achieve satisfactory results. Although the patients’ feedback is a critical aspect, it is relatively subjective. (2) The clinical and imaging examinations. They are relatively reliable but are also affected by the subjective impression of surgeons. (3) Measurement and analysis of imaging data. It’s an objective method as it evaluates the treatment outcomes by the exact measurement of imaging data. In this study, all the above methods were applied to evaluate the accuracy of reductions. The third method is the most important while the first two methods are relatively unreliable. Moreover, in the future projects, we will quantify clinical observation outcomes, such as the evaluation of function and soft tissue symmetry, to demonstrate the clinical significance objectively.

The accuracy of surgical navigation is influenced by various errors, including image conversion, software and hardware products, data registration, and intraoperative procedure^27–29^. Based on our experience, here are some tips to increase the odds of success in the use of surgical navigation.

The first tip is the establishment of the reference plane through the midline. According to previous studies, there has been no standardized guide to establish the reference plane^2,5,30,31^. Marmary *et al*.^30^ and Forsberg *et al*. reported that the midline is based on the neural foramen at the bottom of the skull. Akihiro Ogino *et al*.^5^ reported that the midline is based on the anterior and posterior nasal processes and on the centre of the left and right external acoustic foramens. In this study, we referred to the guide proposed by Feng *et al*.^32^. For those patients with condylar fractures, the reference plane is a sagittal plane through the comb, according to Shen *et al*.^16^. The last and perhaps the most important step in the preoperative plan is to adjust the mirrored image. Because the facial bony contour of any given individual is not perfectly symmetrical, mirrored images must be manually adjusted to fit as precisely as possible to the fixed anatomic landmarks, such as the petrous apex of the temporal bone^19^.

The second tip is the firm installation of the navigation reference frame. The reference frame with 3 light-reflecting balls was fixed with a titanium screw to the patient’s skull, realizing the patient-to-image registration. Once the position of reference frame changes, re-registration is necessary throughout the procedure. Thus, the firm installation of the navigation reference frame must be guaranteed, with any moving or loosening avoided.

The third tip is an accurate registration. Accurate registration is crucial as it has a direct effect on the precision of all subsequent outcomes of operation^33^. Laser surface surgical navigation has been demonstrated to be sufficiently precise in previous studies and was used in this study^34^. With the guidance of surgical navigation, bilateral zygomatical facial regions were scanned in a figure “8” shape. Followed by multipoint verification, performed with a probe to ensure that the patient and operative tools were interconnected. To improve the accuracy, surgeons need to pay attention to the following things. Firstly, movable soft tissues can result in a significant registration error to the clinical precision of laser surface scanning. Therefore, the scanner should be vertical to the skin^29^ and the lateral canthus and nasal root, which is thin and undulating, should be selected to test. Secondly, eye ointment should be chosen to replace eye masks to avoid the distortion and displacement of soft tissue. Thirdly, orotracheal intubation should be chosen to get an undisturbed nasal appearance. Nine patients in the study underwent nasotracheal intubation in the experimental group. Last but not the least, preoperative plan and surgery should be performed as soon as possible after the imaging data of patients were collected to ensure the consistency of preoperative and intraoperative shape of the soft tissue. For patients with obvious swelling, delayed operation and detumescence treatment were sensible. Imaging data were regained after the swelling approximately subsided.

The fourth tip is thorough verification of reduction. The zygomaticomaxillary complex is an irregular-shaped complex, and thus the verification of reduction based on only one or several points is obviously unpersuasive. In this study, a totally scan of the edges of the reduced fragments was performed by the probe and only when the path of probe coincided with the edges of the fragment in the desired position were considered as an accurate reduction.

A concern of use of the navigation system is that surgical virtual plan and navigation process need additional time and cost. As we know, an additional 30 minutes were needed at the beginning of the surgical procedure for installation and fixation of the navigation reference frame, registration process, and recalibration of anatomic landmarks. However, as the results showed, the average preoperative plan time was 48 (32 to 64) minutes vs 46.8 (34 to 64) minutes in the experimental and control groups, respectively. The average surgery time was 183.70 (153 to 229) minutes vs 179.00 (162 to 235) minutes in the experimental and control groups, respectively. There were no significant differences between the groups. We thought the time spent for navigation process during surgery to be more or less compensated by the time saved owing to better orientation and faster reduction. The additional cost for the use of surgical navigation was about 1,000 RMB. Taking treatment outcomes into account, we believe the additional time and cost were acceptable and deserved.

Another concern is whether it causes potential complications of navigation or not. As we know, the application of navigation reference frame is invasive, which may lead to some additional complications, including infection, scarring, or intracranial injury. The results showed that there were no any additional complications related to temporary implantation of this device. Moreover, the scars were minor and invisible in all cases. So, the technology is safe and worthy recommended.

To sum up, we provided evidence that the surgical navigation technique provides an intro-operational guide for surgeons in obtaining accurate reductions. Our findings suggested that the surgical navigation technique is valuable in performing accurate reduction of complicated zygomaticomaxillary complex fractures and restoring facial contour.

## Methods

### Study design and sample

To address the research purpose, the authors designed and conducted a randomized controlled clinical trial (ClinicalTrials.gov ID: NCT03075384, March 8, 2017). The authors read the Declaration of Helsinki and followed these guidelines in this study. All experimental protocols in this clinical prospective study were performed in accordance with CONSORT guidelines and were approved by the Ethics Committee of the West China Hospital of Stomatology, Sichuan University (review document: WCHSIRB-D-2015–016).

The participants were patients with unilateral complicated zygomaticomaxillary complex fractures, undergoing surgery from March 2014 to March 2015 at the Department of Oral and Maxillofacial Surgery at the West China Hospital of Stomatology, Sichuan University. The α = 0.025, 1-β = 90%, σ = 0.6 and the dividing value for clinical significance (δ) = 0.6 were established based on the references and suggestion of clinical specialist. Then, the total number of patients was calculated using PASS19 (IBM Corp, Armonk, NY) software based on the formula n = 2[(u_α_+u_β_)S/δ]^2^, and a total of 17 patients were necessary in this study. Considering the dropout and other reasons, the estimated sample size was expanded by 20%, so that the final sample size was 20 patients. Twenty patients were randomly divided into 2 groups according to a random number table, 10 in each group. Patients in the experimental group were treated with surgical navigation, while the control group was treated without it. All patients signed an informed consent document to participate in this study, and the patients who appeared in the figures provided full permission for their photographs and personal data to be used in medical publications, journals, textbooks, and electronic publications. The data collectors, data analysts and the surgeon were not aware of the allocation. The inclusion criteria were as follows: (1) unilateral complicated zygomaticomaxillary complex fractures, which includes type B with delayed surgery and/or bone defect (complete monofragment zygomatic/tetrapod fracture) or type C (multifragment or comminuted type B zygomatic fracture), as proposed by Zingg *et al*.^35^; (2) conscious patients aged between 18 to 60 years; (3) no contraindications of operation in patients; (4) willing to participate in the study. Cases in which technical problems or registration fails occurred and patients could not complete a 1-year follow-up were excluded from this study.

### Equipment and software

All pre- and postoperative imaging data were obtained (Brilliance CT: 1.0 mm slice thickness, Bright Speed 16, Philips, Netherlands), and then the data were transferred into the Brainlab iPlan CMF 3.0 software (Brainlab, Heimstetten, Germany). With the mirroring tool, the affected side was moved to the target area guided by the healthy side. The Brainlab Vector Vision 2 navigation system (Brainlab, Munich, Germany) was used to assist the operation in the experimental group. Brainlab iPlan CMF 3.0 software and Geomagic Studio 11 software were used to analyse the imaging data.

### Technique for experimental group

#### Preoperative preparation

The preoperative virtual plans were designed with the Brainlab iPlan CMF 3.0 software. We regarded the centre point of the left and right condyle as points A and B, respectively. Point D was the midpoint between point A and B. The sagittal plane was defined as a reference plane through point D and was perpendicular to the line AB. For those patients with condylar fractures, the reference plane is a sagittal plane through the comb. According to the established symmetric plane, the affected side model was obtained by mirroring the healthy side. Then, the mirrored images were manually adjusted to fit the adjacent anatomical structures. The final preoperative virtual plan was collected and saved in digital imaging and communications in Standard Triangle Language (STL) format to help with intraoperative and postoperative analysis.

#### Anaesthesia and maxilla-facial preparation

After routine anaesthesia induction, orotracheal intubation was a preferred anaesthesia. However, patients with occlusal disturbance who needed the occlusal relationship to be reconstructed had to be anaesthetized with nasotracheal intubation. Aureomycin ointment was smeared on the conjunctivae before the operation area was disinfected.

#### Intraoperative navigation

**Figure 1:**
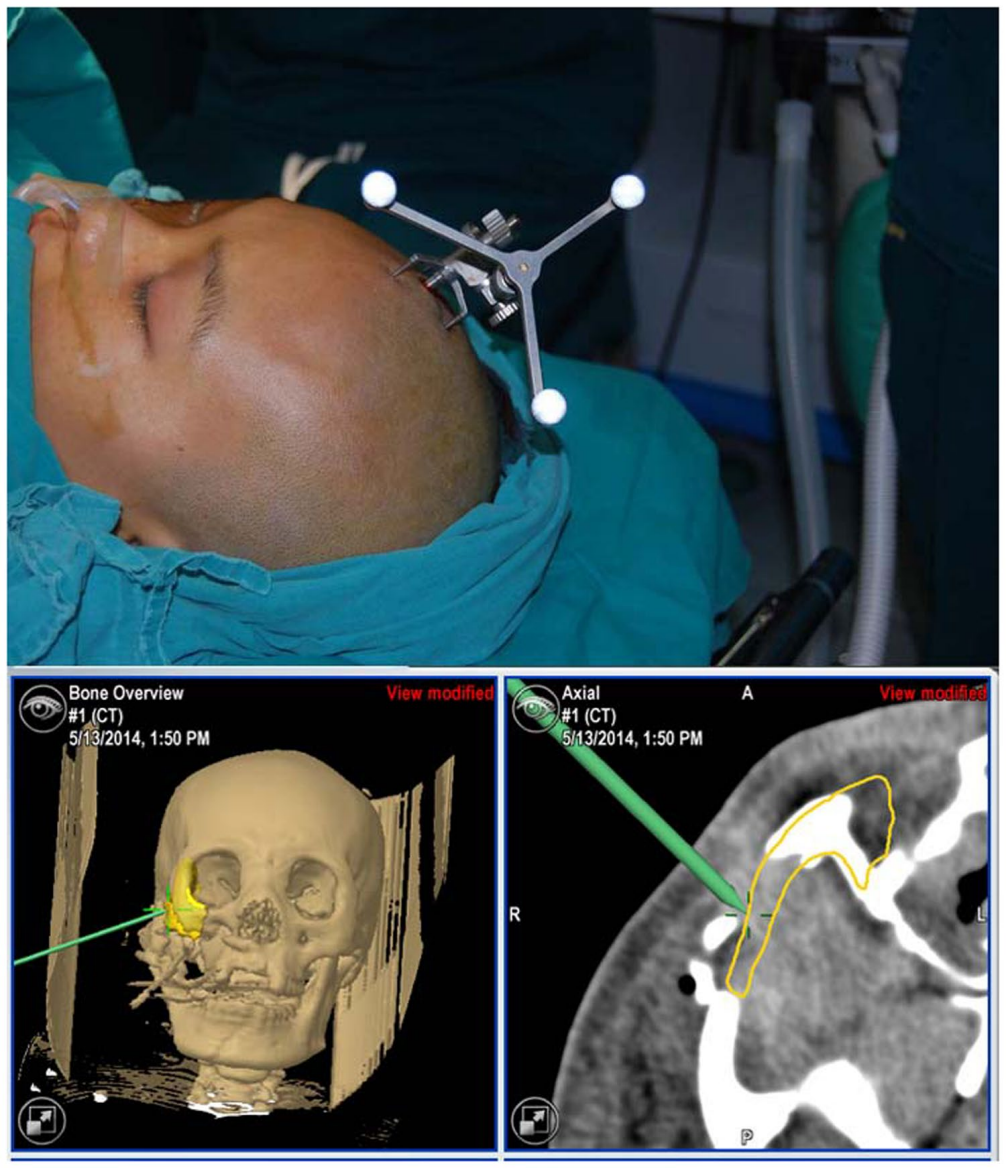
Intraoperative navigation. (**A**) Digital reference frame was fixed; (**B**) Fracture reduction with guidance of surgical navigation.

All operations were performed by one surgeon. The navigation reference frame was installed and fixed firmly on the healthy skull towards the position sensor. Laser surface scanning (z-touch) was used to complete the patient-to-image registration process. The system automatically verified the registration accuracy of the surgical area in all patients, and the registration error was <1.0 mm in all cases. The surgical incisions used varied according to the different situations, including hemicoronary, subconjunctival, intraoral vestibule incision, facial scar, local incision or combined incision. Once the fracture sites were exposed, open reduction was performed. Then, the navigation probe was placed on the edges of the segments to precisely verify the reductions (Fig. 1). After the accurate reduction was verified, internal fixation was applied with titanium plates and screws. An orbital-wall titanium mesh was used for patients with bone defects of the orbital wall.

### Technique for control group

Preoperative preparation, anaesthesia and maxilla-facial preparation in the control group were the same as procedures in the experimental group. Patients were treated surgically, without the assistance of surgical navigation technology, by the same surgeon. Titanium plates or titanium meshes were used to fix the fractured bones.

### Study variables

The primary outcome variable was deviation between pre- and postoperative CT measurement of surgical landmarks. Other variables included age, gender, maximum mouth opening, fracture reduction, wound infection and surgery time.

### Data collection and measurement

Postoperative clinical symptoms and imaging data were collected two weeks after operations in a blinded manner. The patients were followed-up for every 3 months for 1 year to assess and re-evaluate the clinical symptoms and imaging data. The primary outcome variable to evaluate the accuracy of reductions was analysed as follows:

1. The pre- and postoperative imaging data in digital imaging and communications in medicine (DICOM) format were compared using Geomagic Studio 11 software. The matching degree of the pre- and postoperative imaging data was analysed based on the three defined landmarks: oz (the intersection point of the zygomaticomaxillary suture and the infraorbital margin), zm (the most inferior point of the zygomatic maxillary suture) and fmt (the most lateral point of the front zygomatic suture) (Fig. 2). The averages of deviations of these three landmarks were measured with Geomagic Studio 11 software. In this study, one person did all the measurements and measured 3 times for every landmark.

**Figure 2:**
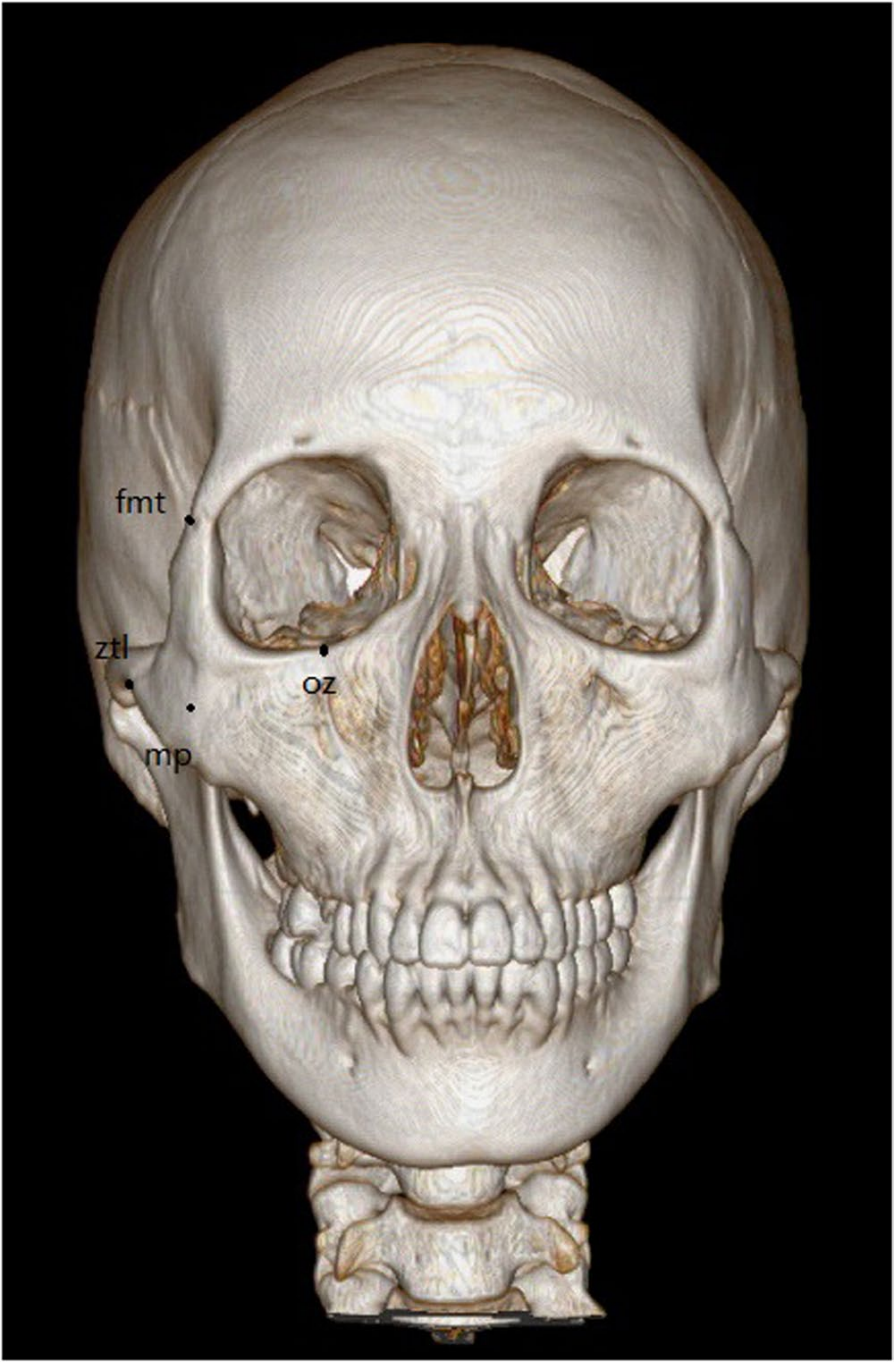
The locations of defined landmarks. oz (the intersection point of the zygomaticomaxillary suture and the infraorbital margin), zm (the most inferior point of the zygomatic maxillary suture), fmt (the most lateral point of the front zygomatic suture), mp (the most anterior point of the zygoma), ztl (the most inferior point of the temporozygomatic suture).
2. The pre- and postoperative imaging data in DICOM format were compared using Brainlab iPlan CMF 3.0 software. The deviations were analysed at four defined landmarks: oz, fmt, mp (the most anterior point of the zygoma), and ztl (the most inferior point of the temporozygomatic suture) (Fig. 2). Deviations of these four landmarks were measured, respectively, with the software for each patient. In this study, one person did all the measurements and measured 3 times for every landmark.

### Statistical analysis

The chi-squared test (Fisher exact probability method) and independent-sample t-test were used for statistical analyses. All statistical analyses were calculated using SPSS 19 for Windows. A P value of less than 0.05 was considered statistically significant.

## Case Reports

**Figure 3:**
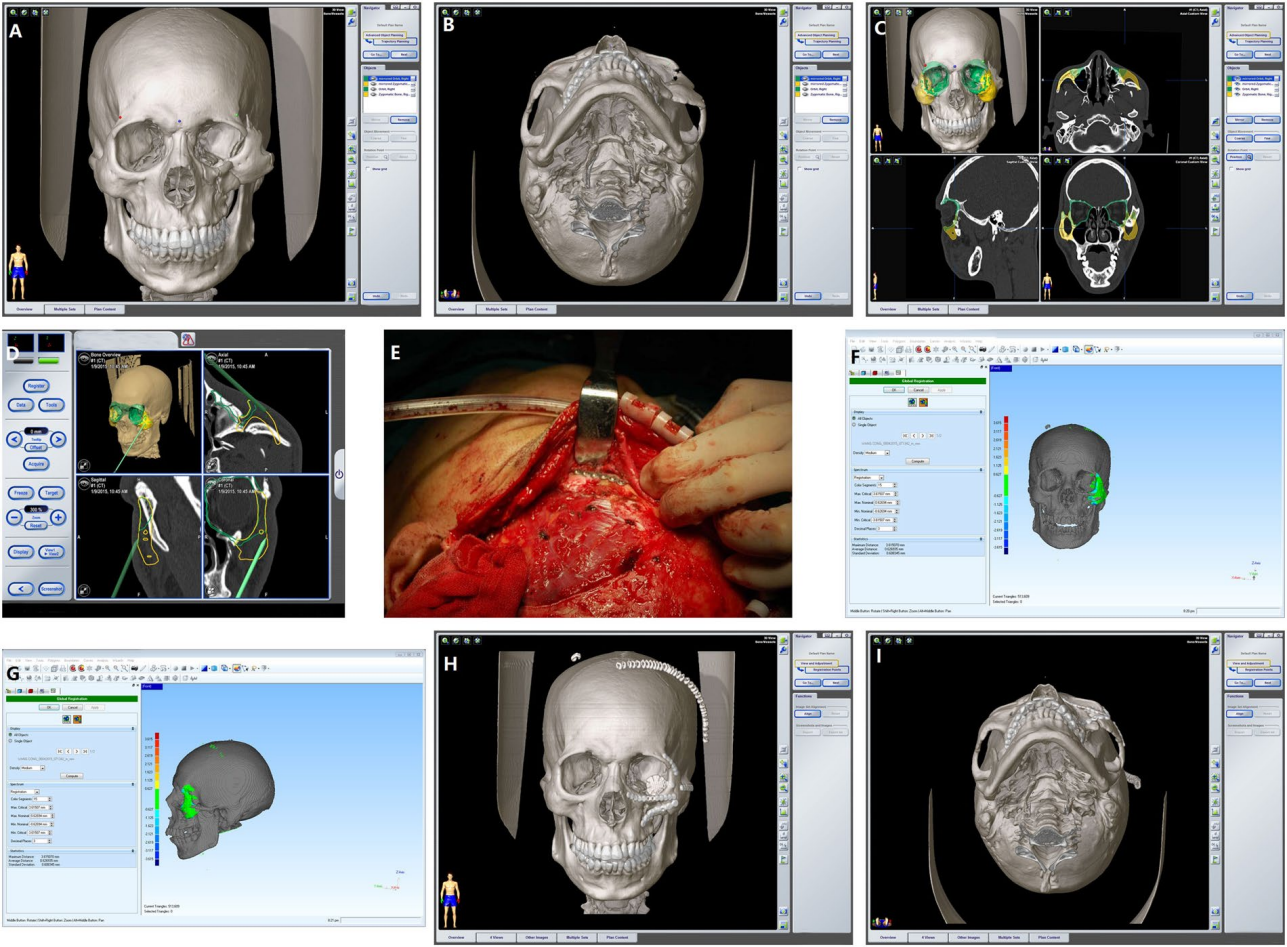
(**A**) 25-year-old man was diagnosed with left complicated zygomaticomaxillary complex fractures and bone defects of left orbital floor. (**A**,**B**) Preoperative imaging data with 3D reconstructions. (**C**) Virtual preoperative reconstruction. (**D**) Reductions of fractures assisted by real-time navigation. (**E**) Internal fixation with titanium plates and screws. (**F**,**G**) The screenshot of data measurement by Geomagic Studio 11 software. (**H**,**I**) Postoperative imaging data with 3D reconstructions.

A 25-year-old man was diagnosed with left complicated zygomaticomaxillary complex fractures and bone defects of left orbital floor. Clinical examination showed the left zygomaticofacial collapse and moderately limited mouth opening. The preoperative surgical plan was accomplished using the Brainlab iPlan CMF 3.0 software. The intraoperative navigation guided reduction of fractures and verified the effect of reductions. The reconstruction of the left orbital floor was also guided by navigation. The fractured bones achieved anatomical reductions. AD, measured by Geomagic Studio 11 software, was 0.627 mm. Postoperative CT examination revealed that fracture reduction was successful, and facial contour was symmetric (Fig. 3).
